# Dynamic transcriptome and network-based analysis of yellow leaf mutant *Ginkgo biloba*

**DOI:** 10.1186/s12870-022-03854-9

**Published:** 2022-09-29

**Authors:** Yue Sun, Pan-Pan Bai, Kai-Jie Gu, Shao-Zong Yang, Han-Yang Lin, Cong-Guang Shi, Yun-Peng Zhao

**Affiliations:** 1 Systematic & Evolutionary Botany and Biodiversity Group, MOE Key Laboratory of Biosystem Homeostasis and Protection, College of Life Sciences, Zhejiang University, Hangzhou, 310058 China; 2 Zhejiang Academy of Forestry, Hangzhou, 310023 China

**Keywords:** Dynamic transcriptome, Leaf yellowing, *Ginkgo biloba*, Golden-leaf cultivar, Physiological feature

## Abstract

**Background:**

Golden leaf in autumn is a prominent feature of deciduous tree species like *Ginkgo biloba* L., a landscape tree widely cultivated worldwide. However, little was known about the molecular mechanisms of leaf yellowing, especially its dynamic regulatory network. Here, we performed a suite of comparative physiological and dynamic transcriptional analyses on the golden-leaf cultivar and the wild type (WT) ginkgo to investigate the underlying mechanisms of leaf yellowing across different seasons.

**Results:**

In the present study, we used the natural bud mutant cultivar with yellow leaves “Wannianjin” (YL) as materials. Physiological analysis revealed that higher ratios of chlorophyll *a* to chlorophyll *b* and carotenoid to chlorophyll *b* caused the leaf yellowing of YL. On the other hand, dynamic transcriptome analyses showed that genes related to chlorophyll metabolism played key a role in leaf coloration. Genes encoding non-yellow coloring 1 (*NYC*1), NYC1-like (*NOL*), and chlorophyllase (*CLH*) involved in the degradation of chlorophyll were up-regulated in spring. At the summer stage, down-regulated *HEMA* encoding glutamyl-tRNA reductase functioned in chlorophyll biosynthesis, while *CLH* involved in chlorophyll degradation was up-regulated, causing a lower chlorophyll accumulation. In carotenoid metabolism, genes encoding zeaxanthin epoxidase (*ZEP*) and 9-cis-epoxy carotenoid dioxygenase (*NCED*) showed significantly different expression levels in the WT and YL. Moreover, the weighted gene co-expression network analysis (WGCNA) suggested that the most associated transcriptional factor, which belongs to the AP2/ERF-ERF family, was engaged in regulating pigment metabolism. Furthermore, quantitative experiments validated the above results.

**Conclusions:**

By comparing the golden-leaf cultivar and the wide type of ginkgo across three seasons, this study not only confirm the vital role of chlorophyll in leaf coloration of YL but also provided new insights into the seasonal transcriptome landscape and co-expression network. Our novel results pinpoint candidate genes for further wet-bench experiments in tree species.

**Supplementary Information:**

The online version contains supplementary material available at 10.1186/s12870-022-03854-9.

## Background

Leaf coloration is a common but essential biological process, especially for ornamental plants and economic crops. Leaf color mutants are ideal materials for exploring the mechanism of leaf coloration, pigment metabolism pathways, chloroplast development, and photosynthetic efficiency, among others [1]. Apart from the physiological changes of leaf color variation, the associated metabolic pathways have also been extensively studied with the development of modern biotechnology in recent years [2].

Leaf color mutant is usually related to the content and relative proportion of various pigments including lipophilic (e.g., chlorophyll and carotenoids) and water-soluble pigments (e.g., anthocyanins). The metabolism process of pigments is regulated by both the environmental and the genetic factors [3]. Temperature and light are critical to the formation of leaf color. The inner leaves of *Brassica campestris* L. cultivar W7–2 were found to turn yellow under low temperatures [4]. In green leaves of *Capsicum annuum* (chili pepper), the carotenoid content is negatively correlated with the light intensity [5]. As for genetic factors, the yellow-green leaf mutant of *Oryza sativa* (rice), which was named *ygl16*, showed lower pigment concentration and photosynthetic capacity compared to the wild type [6]. The yellow leaf variety of *Quercus shumardii* (the Shumard oak) showed a higher ratio of carotenoids to chlorophyll content compared to the wild type. *HEMA* (encoding glutamyl-tRNA reductase) and magnesium chelatase gene *MCH* involved in chlorophyll biosynthesis were also decreased compared to the wild type [7]. In addition, transcriptional factors (TFs) can regulate pigment synthesis by binding to *cis*-acting elements in their target gene promoters to induce or inhibit the expression of structural genes [8]. MYB TFs can combine with bHLH and WD40 protein to form the MBW protein complex, which is the core regulator of anthocyanin synthesis. Genetic transformation using the R2R3-MYB gene was conducted in the study of *Pelargonium cripum*. The transgenic plants showed deep red leaves and higher content of anthocyanins [9]. Yet, the key genes and their regulatory network underlying leaf yellowing in tree mutants have not been clarified.

*Ginkgo biloba* L. (ginkgo, or maidenhair tree), a unique “living fossil” native to China, is a relic component of the warm temperate deciduous broad-leaved forests in East Asia [10, 11]. Nowadays, ginkgo trees are cultivated worldwide as landscape trees for their attractive golden leaves in autumn. Some varieties of ginkgo with constant yellow leaves spanning the whole growth seasons have been observed and bred for commercial industries. These yellow-leaf mutants are also perfect materials for studying mechanisms of leaf yellowing variation. For example, the golden-green striped leaf mutant of ginkgo showed abnormal ultrastructural characteristics of chloroplast and lower chlorophyll contents compared to the normal ginkgo, and the further comparative transcriptional analysis of their leaves collected in May identified 116 differentially expressed genes (DEGs) involved in pigment metabolism and some other related biological processes [12]. Another study of the golden-leaf mutant ginkgo, which focused on leaf color and photosynthetic characteristics, revealed that the maximum net photosynthetic rate, the light compensation point, and the dark respiration rate were significantly enhanced in the mutant leaves [13].

A particular cultivar named Wannianjin (YL) was grafted in 2015. Its leaves appeared yellow in spring when germinated, then turned slightly yellow-green in summer as the temperature rose, and finally returned to golden in autumn. This is significantly different from the normal ginkgo “XHS001” (the wild type, WT), whose leaves remained green in spring and summer and turned yellow in autumn. According to our field observations, the difference was in sharp contrast throughout the year. Thus, we proposed that a dynamic analysis of the metabolism process across the year was promising to understand the underlying regulatory mechanism of leaf yellowing.

In the present study, we focused on seasonal dynamic changes in both the physiological and the molecular aspects between WT and YL during the spring, summer, and autumn stages. We aimed 1) to explore the pigment that affects the coloration of leaf yellowing; 2) to identify the functional genes and TFs involved in pigment metabolism as well as other related biological processes using transcriptome sequencing and reverse transcriptase real-time quantitative polymerase chain reaction (qRT-PCR) experiment; 3) to construct the regulatory networks by performing a weighted gene co-expression network analysis (WGCNA). Our results elucidated the underlying molecular mechanisms of leaf yellowing in golden-leaf ginkgo and promoted the understanding yellow leaf phenology of deciduous tree species. Furthermore, this study provides fundamental guidance for selecting and breeding yellow-leaf ginkgo.

## Results

### Pigment contents of leaves

**Fig. 1:**
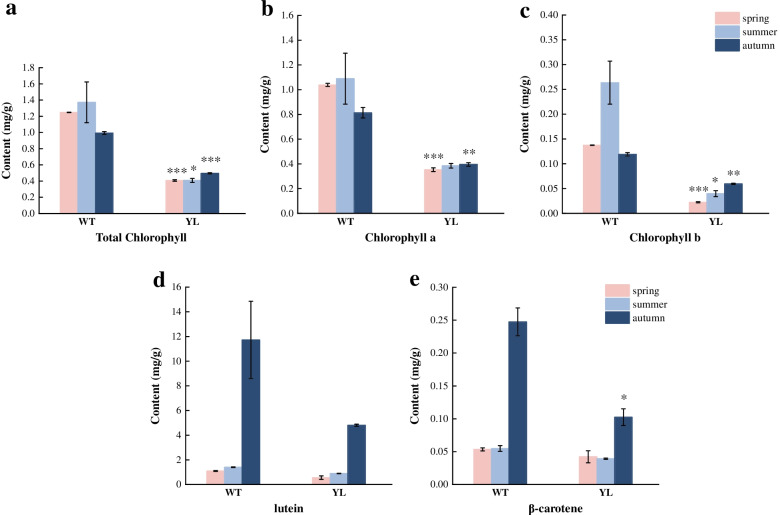
Pigment contents of three stages in wild type (WT) and Wannianjin (YL). **a**–**c.** The chlorophyll content of WT and YL. **d**–**e.** The carotenoid content of WT and YL. Asterisks indicate comparison between WT and YL of the same pigment in the same stage: (*) *P* < 0.05, (**) *P* < 0.01, (***) *P* < 0.001

Based on the phenotypic difference in YL we observed, physiological measurement was performed to investigate the differentiation of pigment contents between WT and YL. Here, the contents of chlorophyll, β-carotene, and lutein in leaves were measured in the spring, summer, and autumn, respectively. For total chlorophyll content, there was no significant change in YL among the three stages, while it decreased in WT from the summer to the autumn stage. The contents of chlorophyll *a* and *b* in YL were significantly lower than in WT at both the spring and autumn stages (Fig. 1a–c). The contents of β-carotene and lutein changed in a similar upward trend in both WT and YL, reaching their peak in autumn. Besides, no significant difference was found between WT and YL in the content of β-carotene or lutein through three stages except for the β-carotene at the autumn stage (Fig. 1d, e). In conclusion, the cultivar YL reduced chlorophyll accumulation, indicating that chlorophyll may have a vital impact on leaf coloration.

To understand the characteristics of pigment content in leaf coloration further, the correlation between chlorophyll *a*, chlorophyll *b*, total chlorophyll, and carotenoid were tested (Table 1). At the spring and summer stages, both the ratios of chlorophyll *a* to chlorophyll *b* and carotenoid to chlorophyll *b* were higher in YL. At the autumn stage, the ratio of carotenoid to chlorophyll *b* increased substantially in YL while that of WT reached a higher value. Other ratios showed no significant difference between WT and YL. Therefore, we proposed that the higher ratios of chlorophyll *a* to chlorophyll *b* and carotenoid to chlorophyll *b* may cause the leaf yellowing of YL.

**Table 1:** Ratio analysis of pigment contents in “XHS001” (WT) and “Wannianjin” (YL) in mg/g fresh weight

Stage	Type	Chlorophyll ***a***/Chlorophyll ***b***	Carotenoid/Chlorophyll ***a***	Carotenoid/Chlorophy ***b***	Carotenoid/Total Chlorophyll
Spring	WT	7.55	1.11	8.38	0.92
	YL	15.69**	1.70	26.66**	1.48
Summer	WT	4.13	1.10	4.54	0.87
	YL	9.66**	1.69	16.28**	1.59
Autumn	WT	6.84	14.71	100.58	12.05
	YL	6.65	12.41	82.52	9.91

Asterisks indicate the significance of the ratio differences between WT and YL at the same stage: (**) *P* < 0.01

### Dynamic transcriptome across seasons

To investigate the gene expression profiles that affect leaf yellowing, transcriptome sequencing of WT and YL at three stages, i.e., the spring, summer, and autumn stages, was performed. About 6.5–9.9 Gb clean data were obtained for each sample after the quality control. The mapping rate of the clean data to the ginkgo genome varied from 80 to 93% (Table S1). Based on the counts, a total of 9303 DEGs were identified between WT and YL (Fig. S1A), of which, 19, 22, and 58.6% were found at the spring, summer, and autumn stages, respectively (spring: 690 up-regulated and 1100 down-regulated; summer: 660 up-regulated and 1404 down-regulated; autumn: 2968 up-regulated and 2481 down-regulated; Table S2). The Gene Ontology (GO) analysis indicated that these DEGs were classified into three categories (Fig. S2) and were significantly enriched (*P* < 0.05) in the “integral component of membrane”, “oxidation-reduction process”, and “nucleus”, among others. For the Kyoto Encyclopedia of Genes and Genomes (KEGG) pathways enrichment analysis, DEGs were assigned to 98, 101, and 120 pathways at three stages, respectively (Fig. S3), such as, “Flavonoid biosynthesis” at the spring stage and “Photosynthesis” at the autumn stage. However, DEGs assigned to “Carotenoid biosynthesis” were not significantly enriched at three stages.

### DEGs involved in chlorophyll metabolism and photosynthesis

**Fig. 2:**
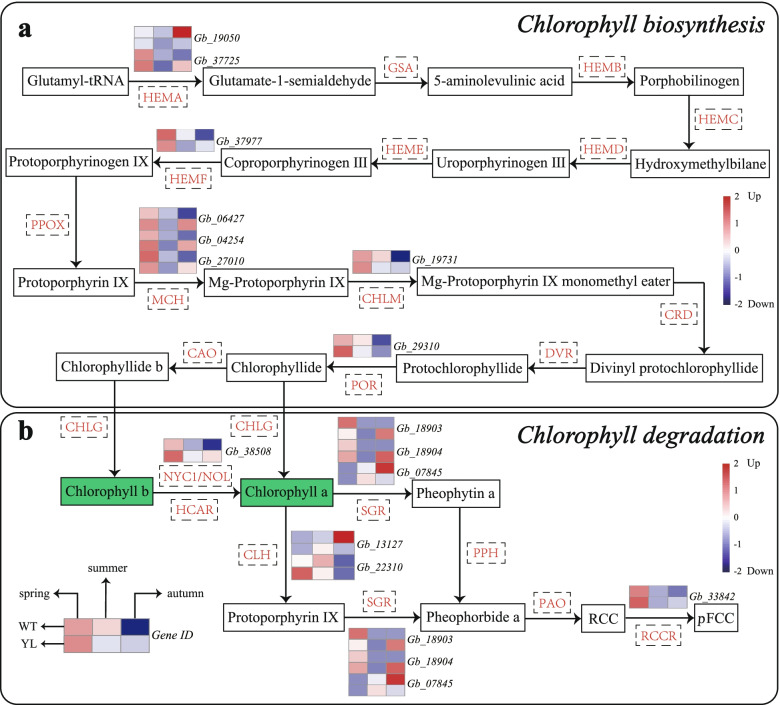
Expression profiles of differently expressed genes (DEGs) involved in chlorophyll metabolism between wild type (WT) and Wannianjin (YL). The expression level was calculated from three biological replications at each stage and scaled by DESeq2. The color bar indicates an increasing expression level from blue to red

The biosynthesis and degradation of chlorophyll play crucial roles in leaf yellowing. 15 DEGs encoding nine enzymes were identified to be associated with chlorophyll metabolism (Fig. 2; Table S3). DEGs involved in the biosynthesis of chlorophyll showed a similar variation trend in WT and YL along the three stages: they all reached the highest at the spring stage and decreased over time (Fig. 2a). However, the expression level of DEGs related to the biosynthesis of chlorophyll in WT and YL during the same stage was different. DEGs in YL showed a lower expression at the summer stage but a higher expression level in autumn than that of WT. As for the chlorophyll degradation pathway, the related genes shared the same changing trend with the chlorophyll biosynthesis pathway (Fig. 2b).

In addition, more than one gene in the biosynthesis and the degradation of chlorophyll were found to be related to the leaf yellowing of YL. At the spring stage, *NYC1/NOL* encoding chlorophyll *b* reductase and *CLH* encoding chlorophyllase (which are involved in degradation) showed higher expression in YL, which may lead to low content of chlorophyll. *HEMA* (which is involved in biosynthesis) was significantly down-regulated at the summer stage, while *CLH* (*Gb_13127*) in degradation was up-regulated. At the autumn stage, a total of seven significantly up-regulated genes in YL were related to chlorophyll biosynthesis, which encodes the enzymes HEMA, HEMF, MCH (including CHLI, CHLD, and CHLH subunits), CHLM, and POR.

**Fig. 3:**
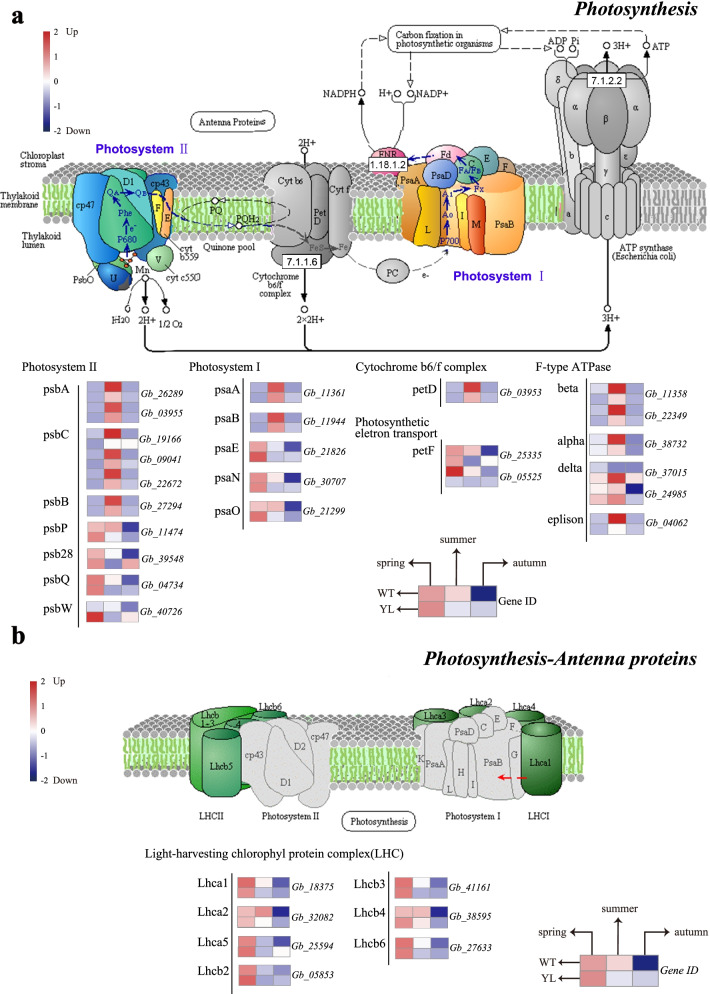
Expression profiles of differently expressed genes (DEGs) involved in photosynthesis between wild type (WT) and Wannianjin (YL). The expression level was calculated from three biological replications at each stage and scaled using DESeq2. The color bar indicates an increasing expression level from blue to red. Schematic illustrations were modified from the KEGG website (https://www.kegg.jp/) [14]

The function and structure of chloroplast are closely related to photosynthesis. There were no genes significantly expressed in the “Photosynthesis” and “Photosynthesis-antenna proteins” pathways between WT and YL at the spring stage. While at the summer stage, seven DEGs were down-regulated in total (Fig. 3, Table S4), indicating the photosynthetic efficiency of YL was lower than that of WT. However, up to 24 DEGs that encode 17 enzymes at the autumn stage showed a higher expression in YL. Generally, it can be safely inferred that leaves of YL had a higher efficiency of photosynthesis in autumn than in summer.

### DEGs involved in carotenoid metabolism

**Fig. 4:**
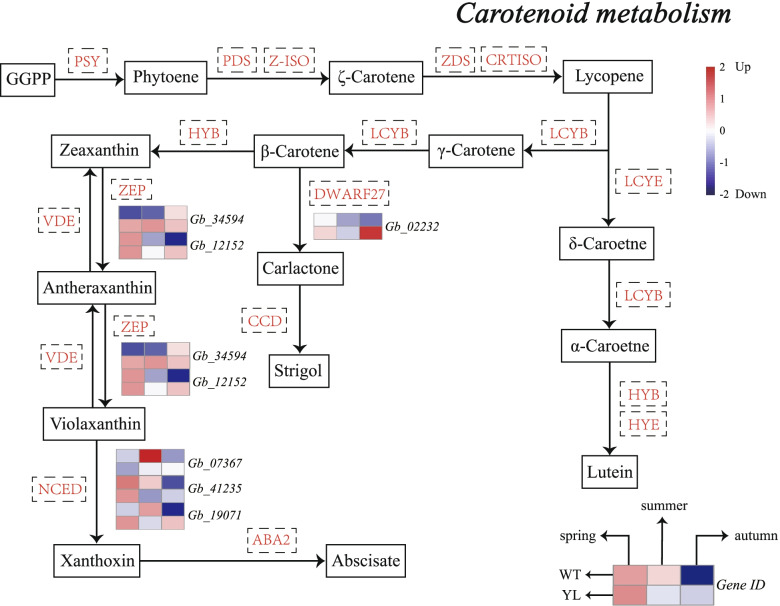
Expression profiles of differently expressed genes (DEGs) involved in carotenoid metabolism between wild type (WT) and Wannianjin (YL). The expression level was calculated from three biological replications at each stage and scaled using DESeq2. The color bar indicates an increasing expression level from blue to red

Carotenoid is a kind of photosynthetic auxiliary pigment that also provides leaves with distinctive yellow and aromas [15]. KEGG analysis showed that six DEGs acted as key genes encoding enzymes related to carotenoid biosynthesis (Table S5). Notably, *ZEP* which encodes zeaxanthin epoxidase was significantly higher in YL than in WT among the three stages (Fig. 4). In addition, the expression of *NCED* which is involved in the degradation of carotenoid was down-regulated in summer but up-regulated in autumn.

### Transcription factors prediction and co-expression network

**Fig. 5:**
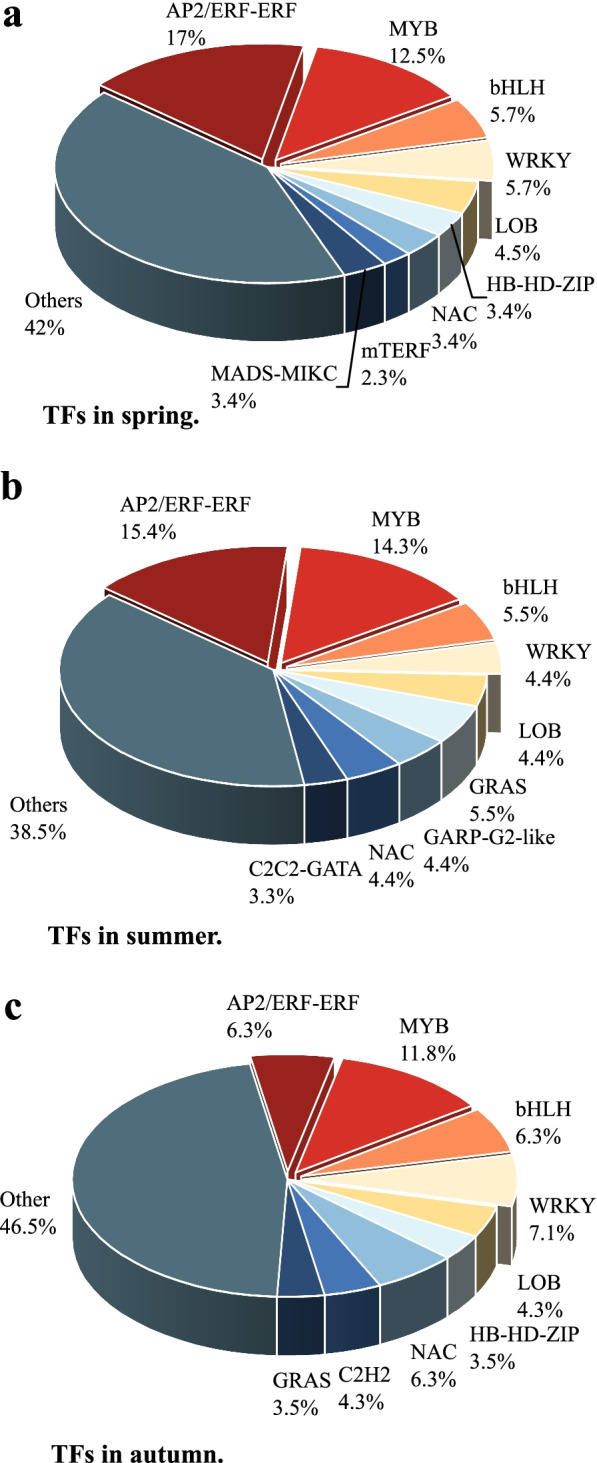
Transcription factors (TFs) that are associated with differentially expressed genes (DEGs) at the spring, summer, and autumn stages. **a** TFs at the spring stage. **b** TFs at the summer stage. **c** TFs at the autumn stage

Transcription factors (TF) play essential regulatory roles in plant development, stress resistance, and secondary metabolism, as well as leaf coloration [9, 16]. Here, 88, 91, and 254 DEGs between WT and YL at three stages were found, belonging to 10, 18, and 42 TF families, respectively. AP2/ERF-ERF, MYB, bHLH, and WRKY were the most abundant TF families at all stages (Fig. 5). At the spring stage, TFs were all highly expressed in YL except for the AP2/ERF-ERF family and all showed lower expression in summer. In autumn, DEGs related to AP2/ERF-ERF, MYB and NAC were up-regulated, while bHLH and WRKY were down-regulated in YL on the contrary (Additional file 2).

**Fig. 6:**
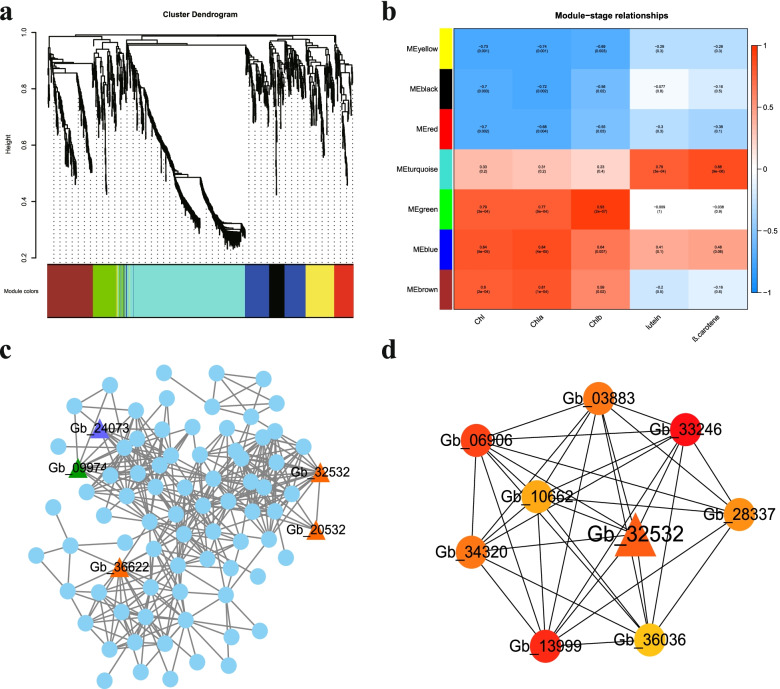
The results of the weighted gene co-expression network analysis (WGCNA). **A** The hierarchical cluster tree shows co-expression modules identified by WGCNA. **B** Module-pigment associations. **C** Correlation networks of genes in the Megreen module. **D** Correlation networks of the top nine hub genes in the Megreen module

To further explore the relationship between TFs and physiological characteristics, a weighted gene co-expression network analysis (WGCNA) was performed to construct potential regulatory networks. Seven distinct modules were identified (labeled by different colors, Fig. 6a). The analysis of the module-trait relationship (Fig. 6b) showed that the content of chlorophyll was highly positively correlated with genes in MEgreen, Meblue, and Mebrown modules (*r* ≥ 0.79) and negatively correlated with Meyellow, Meblack, and Mered modules (*r* ≤ − 0.7). The genes in the Meturquoise module were highly correlated with the content of lutein and β-carotene (*r* ≥ 0.79). The content of chlorophyll *b* showed the highest correlation with the Megreen module, with a coefficient of 0.93 (*P* = 2 × 10^− 7^). Further, the Megreen module which contained 115 genes was visualized by Cytoscape [17], including five TFs (*Gb_32532, Gb_20532, Gb_36622, Gb_24073, Gb_09974*) which belonged to the AP2/ERF-ERF, MYB and SNF2 families (Fig. 6c). The top nine hub genes of Megreen-chlorophyll *b* filtered by cytoHubba [18] contained one gene (*Gb_32532*) belonging to the AP2/ERF-ERF family (Fig. 6d).

### Validation of gene expression by qRT-PCR

**Fig. 7:**
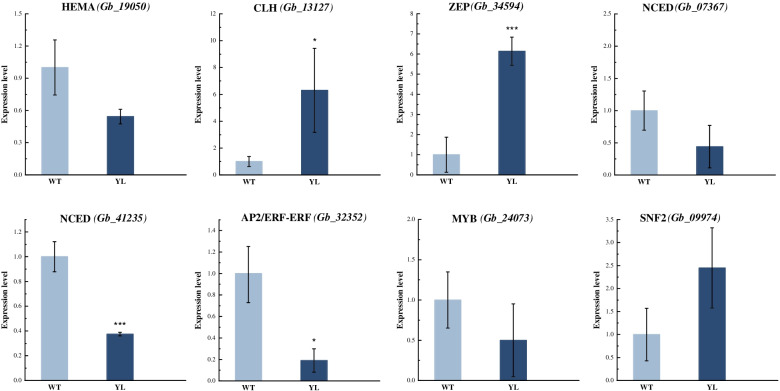
Verification of differently expressed genes (DEGs) between wild type (WT) and Wannianjin (YL) at the summer stage. Asterisks indicate comparison between WT and YL of the same gene: (*) *P* < 0.05, (**) *P* < 0.01, (***) *P* < 0.001

To confirm the accuracy of gene predictions by transcriptomic analysis, we selected the potential DEGs identified above at the summer stage for quantitative real-time RT-PCR (qRT-PCR) analysis. The relative expression levels of eight DEGs: *HEMA, CLH*, *ZEP, NCED*; and DEGs belonging to AP2/ERF-ERF, MYB, and SNF2 families (Fig. 7) were detected. And their expression trends were generally consistent with transcriptomic data. The relative expression of *HEMA* involved in chlorophyll biosynthesis was lower in YL than in WT, while *CLH* involved in chlorophyll degradation was significantly up-regulated in YL. In the pathway of carotenoid metabolism, both *ZEP* in biosynthesis and *NCED (Gb_41235)* in degradation were significantly differentially expressed between WT and YL. In addition, *Gb_32532* belongs to the AP2/ERF-ERF family found through transcripts and the co-expression network showed lower expression in YL than WT. These results confirmed the reliability of the transcriptome data.

## Discussion

### Pigment ratios may determine the leaf yellowing

Leaf coloration is the result of the combined action of different pigments. In our study, the chlorophyll content of YL was significantly lower than that of WT across three seasons, but carotenoid contents showed no significant difference at the green-leaf stage of WT (Fig. 1). Besides, the ratio of chlorophyll *a* to chlorophyll *b*, carotenoid to chlorophyll *b* were higher in YL (Table 1). Similar results in the yellow-green leaf mutant of birch (*Betula platyphylla* × *B. pendula*) showed decreased chlorophyll content and a higher ratio of chlorophyll *a* to chlorophyll *b* compared to the wide type [19]. Moreover, the yellow leaf mutant of *Populus deltoides* showed lower contents of chlorophyll and carotenoids but a higher ratio of carotenoid to chlorophyll compared to normal individuals [20]. Therefore, it can be inferred that the higher ratio of both chlorophyll *a* to chlorophyll *b* and carotenoid to chlorophyll *b* may be important factors in determining the color of golden leaves, and the lower content of chlorophyll *b* can be the main reason for the higher ratios.

### The accumulation of chlorophyll affects photosynthesis and leaf yellowing

There are 16 enzymes encoded by 27 genes involved in the process of chlorophyll biosynthesis from glutamate-tRNA to chlorophyll *a* and *b* [21]. Mutations in any of these genes can lead to the accumulation of intermediates in this pathway and further affect the leaf color. According to the transcriptome data, *HEMA* (which interfered with the synthesis of 5-aminolevulinic acid) was down-regulated in YL at the summer stage, which was also supported by the qRT-PCR, suggesting it was a committed step in chlorophyll biosynthesis (Fig. 2a, Fig. 7). Previous studies in *Arabidopsis thaliana* and *Lagerstroemia indica* both supported that the expression level of *HEMA* was significantly down-regulated in yellow leaf mutants [2, 22]. In addition, DEGs encoding the three subunits of MCH showed lower expression levels in summer. The study of the golden-green striped leaf mutant of ginkgo had a similar result that expressions of all these three genes were down-regulated [12]. Besides, in *Pisum sativum*, *CHLI* and *CHLD* were suppressed by virus-induced gene silencing, both resulting in yellow leaf phenotypes with reduced Mg-chelatase activity [23]. In our study, they were significantly up-regulated at the autumn stage. Therefore, it implies that the efficiency of chlorophyll biosynthesis in YL may be higher than normal ginkgo in autumn.

Degradation is also crucial to the accumulation of chlorophyll. It starts with the reduction of chlorophyll *b*, which is then converted into chlorophyll *a* via two steps of enzymatic reaction [24]. The key enzymes are chlorophyll *b* reductase NYC1 and its homolog, NOL. Moreover, chlorophyllase CLH catalyzes the hydrolysis of chlorophyll to chlorophyllide, and then Mg is removed by Mg-dechelatase SGR (short for STAY-GREEN), which was a chloroplast-located protein and uncovered by screening for stay-green mutants in many species [25]. In this study, *NYC1/NOL* was slightly up-regulated at the spring stage and *CLH* showed significantly higher expression in YL at the summer stage (Fig. 2b). And the relative expression level of *CLH* was verified by qRT-PCR analysis (Fig. 7). Similarly, the higher expression of *CLH* caused yellow leaves in *Populus deltoides* [20].

In summary, these results showed that chlorophyll accumulation was highly regulated by the coordinated transcriptional activation of chlorophyll metabolism genes. At the stage of green-leaf in normal ginkgo, low expression of *HEMA* affected biosynthesis and high expression of *CLH* accelerated degradation of chlorophyll, eventually causing low chlorophyll accumulation (Fig. 1a–c, Fig. 2). Meanwhile, at the autumn stage, the main regulating genes which influenced chlorophyll content were *HEMA, MCH, NYC1/NOL, CLH, SGR,* and so on. However, it differs from a previous report on the golden-green striped leaf mutant of ginkgo, which concluded that *PPO* encoding protoporphyrinogen IX oxidase, *MCH,* and *NYC1/NOL* were the key genes in the chlorophyll metabolism pathway. On the contrary, *HEMA* was up-regulated in the mutant in Li et al.’s (2018) study [12].

In the process of photosynthesis, light energy is captured by pigments in the LHC proteins and transferred to the reaction centers of the thylakoid membrane [26]. Photosystem I (PSI) is a multiprotein complex in the thylakoid membrane and mediates light-driven electron transfer from plastocyanin to Fd [27]. Photosystem II (PSII) is a unique complex capable of absorbing light and splitting water [28]. More than 60% of chlorophyll in plants binds to these two complex systems [29]. In the current study, almost all the DEGs involved in photosynthesis showed a higher expression level at the spring and summer stages than at the autumn stage for both WT and YL, which can be readily understood (Fig. 3). At the summer stage, there were seven DEGs related to the PSII reaction center and F-type ATPase showed reduced expression levels in YL. Therefore, the decreased photosynthesis in YL in summer was likely due to the low expression level of the PSII reaction center. A previous study in a ginkgo yellow leaf mutant also advocated that low expressions of DEGs in PSII were the reason for reduced photosynthetic capacities [12]. Furthermore, there were seven DEGs associated with the LHC gene family and 17 DEGs related to PSI and PSII reaction center significantly up-regulated in YL at the autumn stage. It can be inferred that photosynthetic capacity decreased evidently from summer to autumn in normal ginkgo; but for YL, it changed more slightly and even showed enhanced expression of some genes. Thus, we proposed that YL was less sensitive to temperature fluctuation.

### Gene *ZEP* involved in carotenoid metabolism may play a crucial role in leaf yellowing

Carotenoids are essential natural pigments, which also act as antenna pigments and photo protectors in photosynthesis. The excessive accumulation of carotenoids usually leads to yellow leaf mutants in plants [30]. In our study, there was no significant difference between WT and YL in physiological behaviors (Fig. 1d, e). However, six DEGs related to carotenoid biosynthesis were identified, among which *ZEP* always showed higher expression levels in YL throughout the three stages (Fig. 4). Moreover, the quantitative experiment confirmed the higher expression level in YL (Fig. 7). In most cases, high expression of *ZEP* accelerated the conversion of β-carotene to antheraxanthin and violaxanthin, indicating that it was involved in the downstream pathway of β-carotene biosynthesis. In the study of *Quercus shumardii*, *ZEP* significantly increased in yellow leaves [7]. It was worth noting that in the pigment determination, we only measured the content of β-carotene and lutein, which can explain the contradictory results of carotenoid content in YL. As for the degradation, *NCED* was found to be lower expressed at all stages. NCED was a fundamental enzyme in carotenoid cleavage to indirectly produce abscisic acid in the plant and it was cloned from a variety of plants [31].

### Transcription factors regulating the leaf coloration

TFs are essential factors to regulate the biological process and plant growth [32–35]. The most enriched DEGs among TFs in our study were identified as AP2/ERF-ERF genes. Also, in the co-expression network analysis, an AP2/ERF-ERF gene (*Gb_32532*) was identified as the hub gene in the Megreen-chlorophyll *b* cluster (Fig. 6) and its homolog in *Arabidopsis* is ethylene response factor 110, playing a role in transcription and transcriptional regulation [36]. The experimental verification also supported the result. The AP2/ERF-ERF family contains a large class of transcription factors in plants that mainly participate in abiotic stress responses and are involved in plant development and secondary metabolism [37, 38]. Meanwhile, genes encoding MYB and SNF2 were in the same correlation network. MYB together with bHLH TFs regulate the flavonoid metabolism [39] and they are targeted by long non-coding RNAs in yellow-leaf ginkgo mutants [40]. The SNF2 family is classified as a transcriptional regulator. In plants, SNF2 family proteins work as chromatin remodeling factors that regulate changes in gene expression in response to stress [41]. The Golden2-like (GLK) transcription factors are known to be related to chloroplast development in several plants. *GLK* genes encode a pair of TFs and regulate the expression of photosynthetic nuclear genes [42]. In *Arabidopsis*, the *glk1* and *glk2* double mutants appeared pale green and were deficient in the formation of the photosynthetic apparatus [43]. Nevertheless, *GLK* was not enriched in our study.

## Conclusions

It is the first attempt to investigate the dynamic processes of ginkgo leaf yellowing with both physiological and transcriptomic data. In the present study, we collected leaves of normal ginkgo (WT) and the golden-leaf cultivar (YL) across different seasons to develop an ideal studying system. Based on the results, we conclude that the ratio of chlorophyll *a* to chlorophyll *b*, and the ratio of carotenoid to chlorophyll *b* are the potential key determinants of leaf yellowing. The high ratios in the golden-leaf cultivar during the summer stage of ginkgo can be caused by low chlorophyll accumulation, which results from both decreased expression of biosynthesis gene, i.e., *HEMA*, and increased expression of degradation gene–*CLH.* And *ZEP* and *NCED* involved in carotenoid metabolism also played a considerable part. Additionally, the most related TFs (encoded by *Gb_32532*) in the co-expression network belonged to AP2/ERF-ERF family which participates in plant secondary metabolism. This study focusing on three stages provides a possible explanation of both physiological and molecular aspects of the leaf yellowing mechanism, which lays the foundation for further functional research on leaf yellowing.

## Methods

### Plant materials

The normal cultivar “XHS001” (WT) of *Ginkgo biloba* and the natural bud mutant cultivar with yellow leaves “Wannianjin” (YL) were sampled in this study. All studied trees were grown under the same environmental conditions at the Zhejiang Academy of Forestry in Hangzhou, China. According to the observation of the change of leaf color for YL, we collected leaves with three biological replicates at the spring stage (April), the summer stage (June), and the autumn stage (November) in 2016. Samples were immediately frozen in liquid nitrogen at the field and stored at − 80 °C until use.

### Chlorophyll and carotenoid determination

Approximately 0.2 g leaf from WT and YL were cut into pieces and put into the mortar. A small amount of quartz sand, CaCO_3,_ and 95% ethanol were added to grind the leaf materials into homogenate till the tissues turned white. Then, we filtered the homogenate to obtain the extract which was measured spectrophotometrically at 663, 645, and 652 nm. The carotenoid content was determined using the reversed-phase high-performance liquid chromatography method following Liu [44]. The significance of content differences was determined with a student’s *t*-test.

### RNA extraction and Illumina sequencing

Three biological replications of both WT and YL were prepared for transcriptome sequencing. The total RNA was extracted using the TRIzol Reagent (Invitrogen, Shanghai, China). The quantity and quality of each RNA sample were assessed using agarose gel electrophoresis and the concentration was measured using the NanoDrop (NanoDrop, Wilmington, USA). The integrity of RNA was checked with Agilent 2100 (Agilent, Santa Clara, USA). The construction and sequencing of the cDNA library were performed at Novogene (Beijing, China) using Illumina HiSeq X Ten sequencing platform (Illumina, San Diego, USA). To obtain clean data, adapter sequences and low-quality reads were removed from raw reads. Then the clean reads were aligned to a *Ginkgo biloba* reference genome (https://db.cngb.org/search/assembly/CNA0000042/) using HISAT2 (ver. 2.2.0) [45]. For gene expression analysis, read counts of each gene were calculated by featureCounts (ver. 2.0.0) [46].

### Identification and functional analysis of differentially expressed genes (DEGs)

The R package DESeq2 (ver. 1.26.0) [47] was used to identify genes that were differentially expressed between WT and YL. The gene expression level was normalized by DESeq2 with a negative binomial distribution. Genes with false discovery rate (FDR) ≤ 0.05 and |log_2_fold change (FC)| > 1 were considered as DEGs between samples. DEGs were annotated using BLASTx (ver. 2.9.0+) and diamond (ver. 0.9.31) [48] with a threshold of *E-value* ≤ 1e^− 5^ against the NCBI non-redundant protein sequences (NR). For functional annotation, DEGs were identified by Gene Ontology (GO) and Kyoto Encyclopedia of Genes and Genomes (KEGG) analysis. GO annotation and enrichment analysis was performed by Blast2GO (ver. 5.2) [49]. KEGG annotation was conducted by using KEGG Automatic Annotation Server (KAAS, https://www.genome.jp/tools/kaas/, ver. 2.1) [50] and pathway enrichment was implemented using KOBAS (http://kobas.cbi.pku.edu.cn/kobas3, ver. 3.0) [51] with a corrected *p*-value ≤0.05. The transcription factors and transcriptional regulators were predicted using iTAK (http://itak.feilab.net/cgi-bin/itak/index.cgi, ver. 18.12) [52].

### Weighted gene co-expression network analysis

The R package WGCNA (ver. 1.69) [53] was used to construct a co-expression network and to identify the modules of highly correlated genes based on the normalized expression matrix of DEGs. The modules were obtained using the automatic network construction function blockwise with default settings, except that the soft power was set to 12, the minimal module size was set to 30, and the merge cut height was set to 0.25. The networks of candidate target genes were visualized by Cytoscape (ver. 3.8.2) [17].

### RNA extraction and qRT-PCR

Total RNAs were extracted from leaves of WT and YL (three biological replicates) at the summer stage (July) using a plant RNA isolation kit (Vazyme, Nanjing, China). First-strand cDNA was synthesized from 0.5 μg of total RNA using RevertAid Reverse Transcriptase (Thermo Scientific, Waltham, USA) with oligo(dT) primers. The gene *GbACT* of ginkgo was used as an internal reference gene [54]. qPCR analysis was performed using a RealPlex real-time PCR machine (Eppendorf, Hamburg, Germany). The PCR cycles were first denatured (95 °C/2 min), followed by 40 cycles of 95 °C/15 s, 55 °C/15 s, 72 °C/20 s, and finally 1 cycle of 95 °C/15 s, 60 °C/15 s, 95 °C/15 s. Relative expression levels of target genes were calculated with the 2^−ΔΔCt^ comparative Ct method [55]. The primers used are listed in Table S6.

## Supplementary Information

**Additional file 1: Table S1.** Summary of the sequencing and mapping results. **Table S2.** The number of differentially expressed genes in the wild type (WT) and Wannianjin (YL). **Table S3.** The Kyoto Encyclopedia of Genes and Genomes (KEGG) annotation of differently expressed genes in chlorophyll metabolism. **Table S4.** The Kyoto Encyclopedia of Genes and Genomes (KEGG) annotation of differently expressed genes in photosynthesis. **Table S5**. The Kyoto Encyclopedia of Genes and Genomes (KEGG) annotation of differently expressed genes in carotenoid metabolism. **Table S6**. Quantitative real-time PCR primers of differentially expressed genes. **Fig. S1.** The differentially expressed genes between wild type (WT) and Wannianjin (YL) at the spring, summer, and autumn stages, respectively. **Fig. S2.** The most enriched Gene Ontology terms of differentially expressed genes between wild type (WT) and Wannianjin (YL) at the spring, summer, and autumn stages, respectively. **Fig. S3.** The Kyoto Encyclopedia of Genes and Genomes (KEGG) annotation of differentially expressed genes between wild type (WT) and Wannianjin (YL) at the spring, summer, and autumn stages, respectively.

**Additional file 2.** Transcription Factors annotation.

## Data Availability

The datasets generated during the current study are available in the GinkgoDB repository, https://ginkgo.zju.edu.cn/project/T0002/ and also available in the Genome Sequence Archive (Genomics, Proteomics & Bioinformatics 2021) in National Genomics Data Center (Nucleic Acids Res 2022), China National Center for Bioinformation / Beijing Institute of Genomics, Chinese Academy of Sciences (GSA: CRA006977) that are publicly accessible at https://ngdc.cncb.ac.cn/gsa.
