# Supplementary material for: Dynamic transcriptome and network-based analysis of yellow leaf mutant Ginkgo biloba
Source: BMC Plant Biol. 2022 Sep 29;22:465. doi: 10.1186/s12870-022-03854-9 (PMC9520803; doi:10.1186/s12870-022-03854-9)
Supplement: Supplementary file 1 — Additional file 1: Table S1. Summary of the sequencing and mapping results. Table S2. The number of differentially expressed genes in the wild type (WT) and Wannianjin (YL). Table S3. The Kyoto Encyclopedia of Genes and Genomes (KEGG) annotation of differently expressed genes in chlorophyll metabolism. Table S4. The Kyoto Encyclopedia of Genes and Genomes (KEGG) annotation of differently expressed genes in photosynthesis. Table S5. The Kyoto Encyclopedia of Genes and Genomes (KEGG) annotation of differently expressed genes in carotenoid metabolism. Table S6. Quantitative real-time PCR primers of differentially expressed genes. Fig. S1. The differentially expressed genes between wild type (WT) and Wannianjin (YL) at the spring, summer, and autumn stages, respectively. Fig. S2. The most enriched Gene Ontology terms of differentially expressed genes between wild type (WT) and Wannianjin (YL) at the spring, summer, and autumn stages, respectively. Fig. S3. The Kyoto Encyclopedia of Genes and Genomes (KEGG) annotation of differentially expressed genes between wild type (WT) and Wannianjin (YL) at the spring, summer, and autumn stages, respectively. [file 12870_2022_3854_MOESM1_ESM.docx]

# Supplementary material

**Table S1 Summary of the sequencing and mapping results.**

|  | Sample | Raw Reads | Clean reads | Clean bases | Error(%) | Q20(%) | GC(%) | Mapping(%) |
| --- | --- | --- | --- | --- | --- | --- | --- | --- |
| Spring | WTa1 | 53505946 | 51772850 | 7.77G | 0.02 | 96.37 | 46.12 | 89.44 |
|  | WTb1 | 61249794 | 59246560 | 8.89G | 0.02 | 96.37 | 46.00 | 89.07 |
|  | WTc1 | 51535772 | 49795598 | 7.47G | 0.02 | 96.31 | 46.10 | 88.94 |
|  | YL11 | 61711340 | 59640006 | 8.95G | 0.02 | 96.33 | 46.29 | 89.02 |
|  | YL21 | 52885680 | 51176080 | 7.68G | 0.02 | 96.39 | 45.87 | 88.88 |
|  | YL31 | 56744042 | 54826070 | 8.22G | 0.02 | 96.47 | 46.15 | 89.20 |
| Summer | WTa2 | 53242644 | 50763964 | 7.61G | 0.02 | 97.01 | 47.15 | 85.82 |
|  | WTb2 | 45554798 | 43427610 | 6.51G | 0.02 | 96.15 | 47.17 | 82.01 |
|  | WTc2 | 69633768 | 66188428 | 9.93G | 0.02 | 96.30 | 46.84 | 83.78 |
|  | YL12 | 54967324 | 52511954 | 7.88G | 0.02 | 96.65 | 46.01 | 85.93 |
|  | YL22 | 52277738 | 49691478 | 7.45G | 0.02 | 96.79 | 46.28 | 85.14 |
|  | YL32 | 58250122 | 55567268 | 8.34G | 0.02 | 96.57 | 46.25 | 85.57 |
| Autumn | WTa3 | 45701920 | 44391362 | 6.66G | 0.01 | 97.24 | 44.51 | 93.06 |
|  | WTb3 | 47736170 | 45615458 | 6.84G | 0.01 | 98.39 | 45.44 | 90.47 |
|  | YL13 | 65859422 | 63777394 | 9.57G | 0.02 | 96.80 | 46.36 | 90.10 |
|  | YL23 | 59940918 | 57747440 | 8.66G | 0.02 | 97.02 | 45.08 | 92.70 |

**Table S2 The number of differently expressed genes in wild type (WT) and Wannianjin (YL).**

| DEG set | DEG number | Up-regulated | Down-regulated |
| --- | --- | --- | --- |
| Spring | 1790 | 690 | 1100 |
| Summer | 2064 | 660 | 1404 |
| Autumn | 5449 | 2968 | 2481 |

**Table S3** **The Kyoto Encyclopedia of Genes and Genomes (KEGG) annotation of differently expressed genes in chlorophyll metabolism.**

| **Function** | **Gene ID** | **Enzyme** | **Expression level in mutant** | **Annotation** |
| --- | --- | --- | --- | --- |
| Chlorophyll metabolism  (summer) | *Gb_19050* | HEMA | Down-regulated | Glutamyl-tRNA reductase |
|  | *Gb_13127* | CLH | Up-regulated | Chlorophyllase |
| Chlorophyll metabolism  (autumn) | *Gb_37725* | HEMA | Up-regulated | Glutamyl-tRNA reductase |
|  | *Gb_37977* | HEMF | Up-regulated | Coproporphyrinogen III oxidase |
|  | *Gb_06427* | MCH | Up-regulated | Mg-chelatase H subunit |
|  | *Gb_04254* | MCH | Up-regulated | Mg-chelatase D subunit |
|  | *Gb_27010* | MCH | Up-regulated | Mg-chelatase I subunit |
|  | *Gb_19731* | CHLM | Up-regulated | Magnesium proto IX methyltransferase |
|  | *Gb_29310* | POR | Up-regulated | Protochlorophyllide oxidoreductase |
|  | *Gb_38508* | NYC1/NOL | Up-regulated | Chlorophyll(ide) b reductase |
|  | *Gb_13127* | CLH | Down-regulated | Chlorophyllase |
|  | *Gb_22310* | CLH | Up-regulated | Chlorophyllase |
|  | *Gb_07845* | SGR | Down-regulated | Magnesium dechelatase |
|  | *Gb_18903* | SGR | Up-regulated | Magnesium dechelatase |
|  | *Gb_18904* | SGR | Up-regulated | Magnesium dechelatase |
|  | *Gb_33842* | RCCR | Up-regulated | Red chlorophyll catabolite reductase |

**Table S4** **The Kyoto Encyclopedia of Genes and Genomes (KEGG) annotation of differently expressed genes in photosynthesis.**

| **Function** | **Gene ID** | **Enzyme** | **Expression level in mutant** | **Annotation** |
| --- | --- | --- | --- | --- |
| Photosynthesis（summer） | *Gb_19166* | psbC | Down-regulated | photosystem II CP43 chlorophyll apoprotein |
|  | *Gb_25335* | petF | Down-regulated | ferredoxin |
|  | *Gb_04062* | atpC | Down-regulated | F-type H+-transporting ATPase subunit epsilon |
|  | *Gb_11358* | atpD | Down-regulated | F-type H+/Na+-transporting ATPase subunit beta |
|  | *Gb_22349* | atpD | Down-regulated | F-type H+/Na+-transporting ATPase subunit beta |
|  | *Gb_38732* | atpA | Down-regulated | F-type H+/Na+-transporting ATPase subunit alpha |
|  | *Gb_37015* | atpH | Up-regulated | F-type H+-transporting ATPase subunit delta |
| Photosynthesis（autumn） | *Gb_26289* | psbA | Up-regulated | photosystem II P680 reaction center D1 protein |
|  | *Gb_03955* | psbA | Up-regulated | photosystem II P680 reaction center D1 protein |
|  | *Gb_27294* | psbB | Up-regulated | photosystem II CP47 chlorophyll apoprotein |
|  | *Gb_09041* | psbC | Up-regulated | photosystem II CP43 chlorophyll apoprotein |
|  | *Gb_22672* | psbC | Up-regulated | photosystem II CP43 chlorophyll apoprotein |
|  | *Gb_11474* | psbP | Up-regulated | photosystem II oxygen-evolving enhancer protein 2 |
|  | *Gb_04734* | psbQ | Up-regulated | photosystem II oxygen-evolving enhancer protein 3 |
|  | *Gb_40726* | psbW | Up-regulated | photosystem II PsbW protein |
|  | *Gb_39548* | psb28 | Up-regulated | photosystem II 13kDa protein |
|  | *Gb_11361* | psaA | Up-regulated | photosystem I P700 chlorophyll a apoprotein A1 |
|  | *Gb_11944* | psaB | Up-regulated | photosystem I P700 chlorophyll a apoprotein A2 |
|  | *Gb_21826* | psaE | Up-regulated | photosystem I subunit IV |
|  | *Gb_30707* | psaN | Up-regulated | photosystem I subunit PsaN |
|  | *Gb_21299* | psaO | Up-regulated | photosystem I subunit PsaO |
|  | *Gb_03953* | petD | Up-regulated | cytochrome b6-f complex subunit 4 |
|  | *Gb_25335* | petF | Up-regulated | ferredoxin |
|  | *Gb_24985* | atpH | Up-regulated | F-type H+-transporting ATPase subunit delta |
| Photosynthesis-antenna proteins（autumn） | *Gb_18375* | LHCA1 | Up-regulated | light-harvesting complex I chlorophyll a/b binding protein 1 |
|  | *Gb_32082* | LHCA2 | Up-regulated | light-harvesting complex I chlorophyll a/b binding protein 2 |
|  | *Gb_25594* | LHCA5 | Up-regulated | light-harvesting complex I chlorophyll a/b binding protein 5 |
|  | *Gb_05853* | LHCB2 | Up-regulated | light-harvesting complex II chlorophyll a/b binding protein 2 |
|  | *Gb_41161* | LHCB3 | Up-regulated | light-harvesting complex II chlorophyll a/b binding protein 3 |
|  | *Gb_38595* | LHCB4 | Up-regulated | light-harvesting complex II chlorophyll a/b binding protein 4 |
|  | *Gb_27633* | LHCB6 | Up-regulated | light-harvesting complex II chlorophyll a/b binding protein 6 |

**Table S5** **The Kyoto Encyclopedia of Genes and Genomes (KEGG) annotation of differently expressed genes in carotenoid metabolism.**

| **Function** | **Gene ID** | **Enzyme** | **Expression level in mutant** | **Annotation** |
| --- | --- | --- | --- | --- |
| Carotenoid biosynthesis（spring） | *Gb_34594* | ZEP | Up-regulated | zeaxanthin epoxidase |
| Carotenoid biosynthesis（summer） | *Gb_34594* | ZEP | Up-regulated | zeaxanthin epoxidase |
|  | *Gb_07367* | NCED | Down-regulated | 9-cis-epoxycarotenoid dioxygenase |
|  | *Gb_41235* | NCED | Down-regulated | 9-cis-epoxycarotenoid dioxygenase |
| Carotenoid biosynthesis（autumn） | *Gb_12152* | ZEP | Up-regulated | zeaxanthin epoxidase |
|  | *Gb_19071* | NCED | Up-regulated | 9-cis-epoxycarotenoid dioxygenase |
|  | *Gb_02232* | DWARF27 | Up-regulated | β-carotene isomerase |

**Table S6 Quantitative real-time PCR primers of differently expressed genes.**

| **Gene** | **Gene ID** | **Primers (forward)** | **Primers (reverse)** |
| --- | --- | --- | --- |
| *GbACT* | / | TGAATCCCAAGGCAAATAGAGAG | CCCCAGAATCCAAAACAATACC |
| *NCED* | *Gb_41235* | GCAGCCTTCCTTCATACACG | CCCAATCTCGGCACTTTAGC |
|  | *Gb_07367* | ACGAGCCTCTCAGAAAGCATT | TCGGCGCAATTTCTCTTCTG |
| *SNF2* | *Gb_09974* | AAACATACCCTGCCCGAAGA | TCGAGCTTGGTGACCTTCAT |
| *HEMA* | *Gb_19050* | GAGTTGGATTCGGTCAGGGT | TGCCATGCCTCAAATTGCTT |
| *MYB* | *Gb_24073* | ATGCGACCATCATTCAAGCC | AAGGTAGCGACGTCTAAGGG |
| *AP2/ERF-ERF* | *Gb_32532* | GCGGCATCATGGTATACGTC | ACCTTCCTCCCTCTGTCTCT |
| *ZEP* | *Gb_34594* | ACAGTACGCTTCCCATTCCA | CTGCTGTTTGTGAGGGTACG |
| *CLH* | *Gb_13127* | GCTCAACATACGGCCATCAG | CGTGGTTCAACAGAAAGCCA |


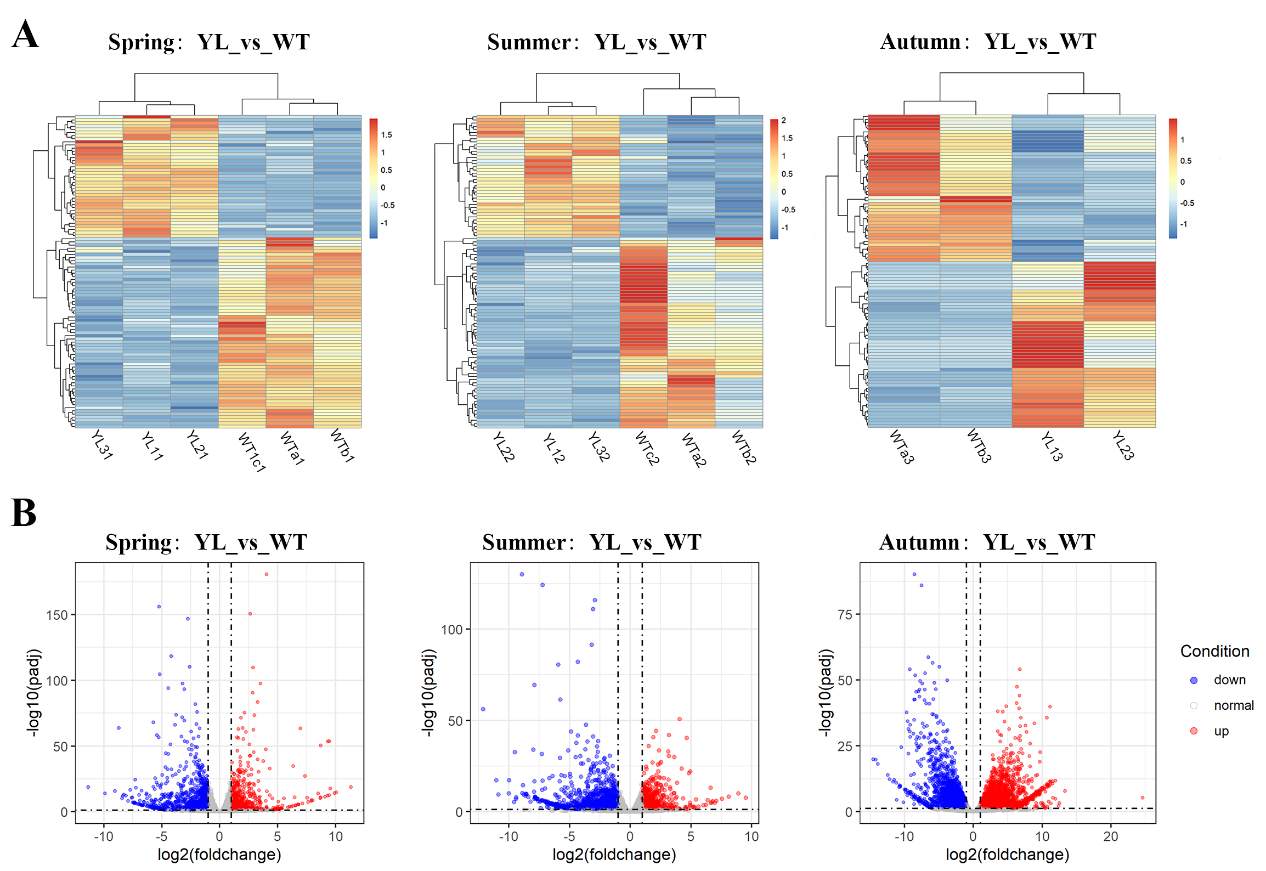


**Figure S1** **The differently expressed genes between wild type (WT) and Wannianjin (YL) at the spring, summer, autumn stages, respectively.**


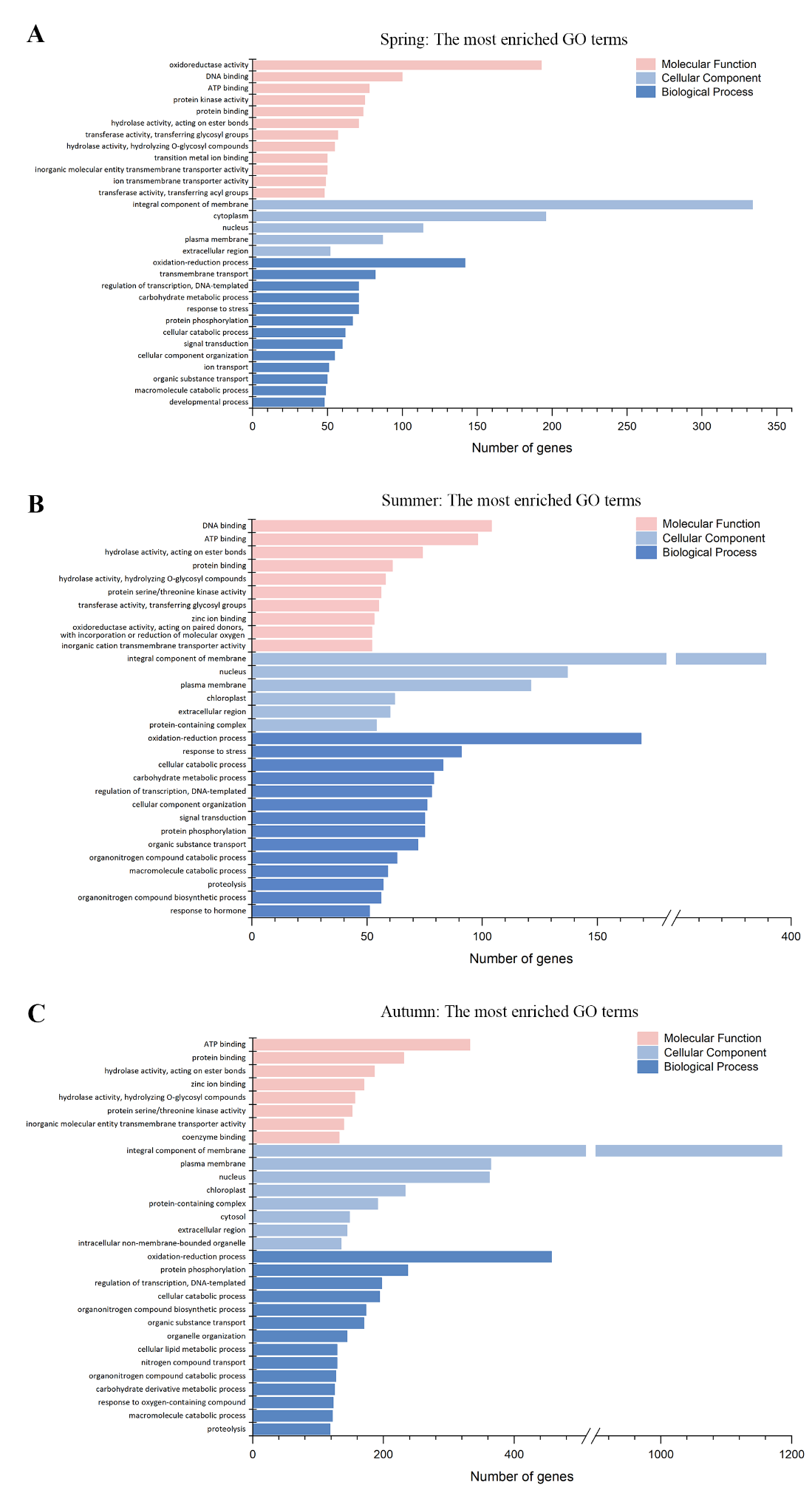


**Figure S2** **The most enriched Gene Ontology terms of differently expressed genes between wild type (WT) and Wannianjin (YL) at the spring, summer, autumn stages, respectively.**


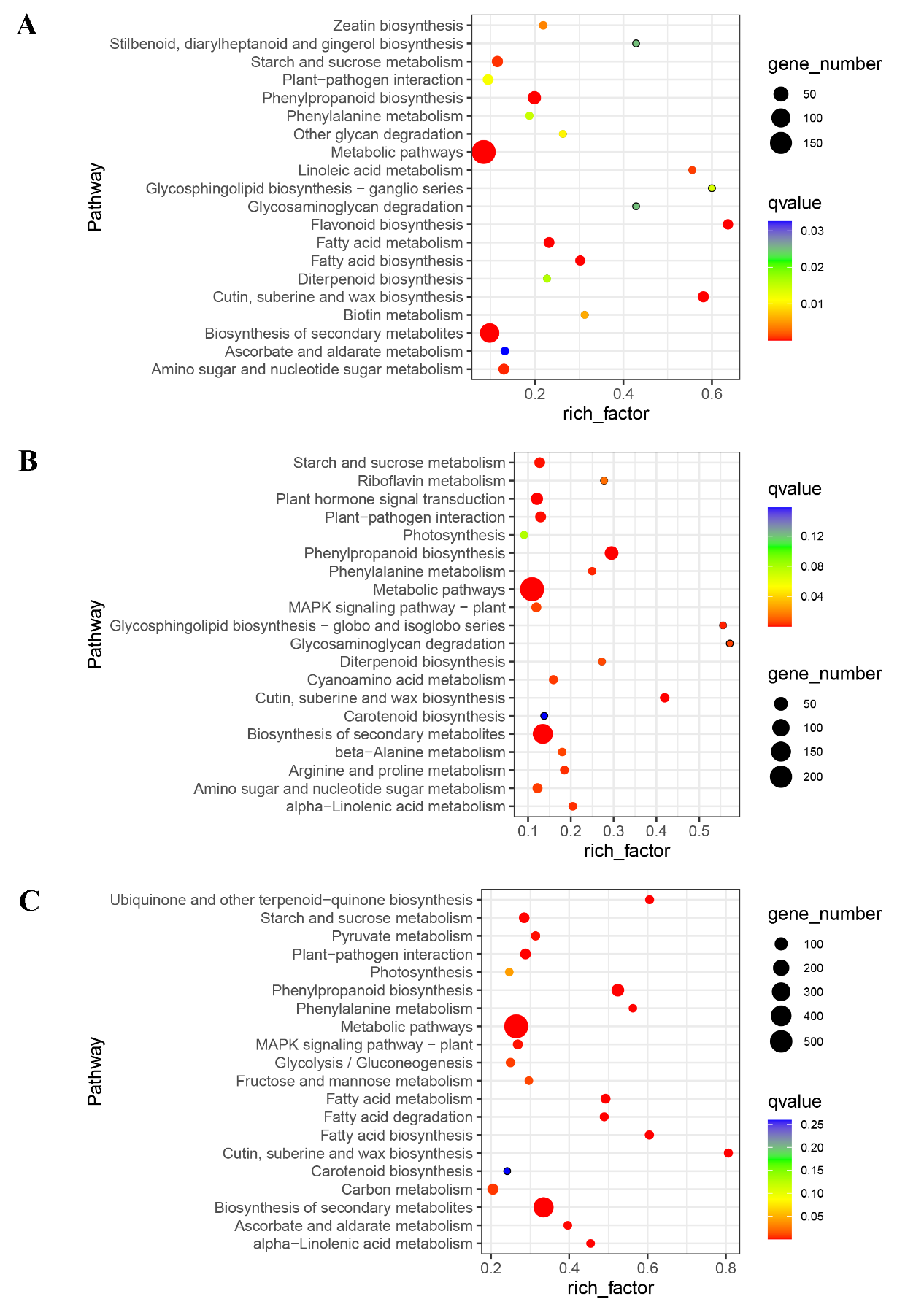


**Figure S3** **The Kyoto Encyclopedia of Genes and Genomes (KEGG) annotation of differently expressed genes (DEGs) between wild type (WT) and Wannianjin (YL) at the spring (a), summer (b), autumn (c) stages, respectively.**
